# Supplementary material for: A membrane-disruptive action of VBIT-4 challenges its role as a widely used VDAC1 oligomerization inhibitor
Source: eLife. 2026 Oct 9;15:RP111980. doi: 10.7554/eLife.111980 (PMC13652541; doi:10.7554/eLife.111980)
Supplement: Supplementary file 2 [file elife-111980-supp1.docx]

| **PLM Lipid**  **composition** | **KCl, M** | **Number of**  **experiments** | **G_m_, pS  10 μM**  **VBIT-4** | **G_m_, pS  20 μM**  **VBIT-4** | **G_m_, pS  50 μM**  **VBIT-4** |
| --- | --- | --- | --- | --- | --- |
| **DPhPC** | 0.15 | 2 | 30 ; 230 | 540 |  |
| **PLE** | 0.15 | 6 | 180 ; 590 | 3200 | 850 ; 1110 |
| **PLE** | 1 | 3 | 29 ; 200 | 316 ; 97 | 3260 |
| **PC:PE:chol** | 0.15 | 3 | 54 ; 45 | 670 ; 806 | 319; 3630 |

**Supplemental Table 1. VBIT-4 induces conductance in planar lipid membranes (PLMs) of different lipid compositions.** Summary of electrophysiology experiments performed on PLMs of three lipid compositions – DPhPC, PLE, and DOPC:DOPE:chol (60:32.5:7.5) - in membrane-bathing solutions of 0.15 or 1.0 M KCl, buffered with 5 mM HEPES at pH 7.4. The effect of VBIT-4 on membrane conductance (G_m_) was measured ~15 min after the addition of VBIT-4 to one side (*cis*) of the membrane at an applied voltage of 100 mV. Each G_m_ corresponds to the highest conductance measured at a given VBIT-4 concentration; those shown in the same color in each row correspond to the G_m_ values measured on the same membrane. G_m_ varies significantly across experiments, even under the same experimental conditions. Some PLMs ruptured upon the addition of 10 µM VBIT-4, while others remained stable until higher concentrations. Most membranes ruptured at ~30 µM of VBIT-4, although a subset resisted up to 50 µM VBIT-4. Notably, 50 µM was the highest concentration of VBIT-4 at which PLMs remained intact for at least 15 min after VBIT-4 addition at an applied voltage of 100 mV. Only the PLMs with conductance < 1 pS in the control before VBIT-4 addition were used in all experiments.
