# Supplementary material for: A membrane-disruptive action of VBIT-4 challenges its role as a widely used VDAC1 oligomerization inhibitor
Source: eLife. 2026 Oct 9;15:RP111980. doi: 10.7554/eLife.111980 (PMC13652541; doi:10.7554/eLife.111980)
Supplement: Peer review history

# Editor report: eLife Assessment

**DOI:** 10.7554/eLife.111980.3.sa0

**Authors:** Fabrizio Marinelli

**Affiliations:** Medical College of Wisconsin United States

**Keywords:** Compelling, Fundamental

This work provides a reassessment of VBIT-4, a compound previously proposed to inhibit oligomerization of the crucial protein known as the mitochondrial voltage-dependent anion channel. Combining complementary experimental approaches with molecular dynamics simulations, the authors provide **compelling** evidence that VBIT-4 primarily disrupts lipid membranes and induces channel-independent cytotoxicity. The study has **fundamental** implications for interpreting previous work using VBIT-4 as a probe of channel function and highlights the need to consider membrane-disruptive effects when evaluating drug mechanisms.

# Referee report: Reviewer #1 (Public review)

**DOI:** 10.7554/eLife.111980.3.sa1

Summary:

The Voltage-Dependent Anion Channel 1 (VDAC1) is the most abundant β-barrel protein in the outer mitochondrial membrane and the main conduit for metabolite and ion exchange between the cytosol and mitochondria. Its oligomerization has been proposed to control mitochondrion-mediated apoptosis, making it a prime target for therapeutic intervention in diseases associated with excessive cell death, such as neurodegenerative disorders and autoimmunity. VBIT-4 is a small molecule developed to inhibit VDAC oligomerization and has shown therapeutic potential in various preclinical models. Despite its widespread use, the mechanism of action of VBIT-4 has not yet been fully elucidated. In this paper, Ravishankar et al. combine a suite of biophysical approaches with computer simulations to demonstrate that VBIT-4 forms water-permeable defects in membrane bilayers without any detectable effects on VDAC1 channel properties or oligomerization. Furthermore, cytotoxicity assays revealed identical VBIT-4 IC50 values in wild-type and VDAC1-KO cells, indicating that its activity does not depend on VDAC1. Collectively, these findings cast significant doubt on the widely held assumption that VBIT-4 is a specific inhibitor of VDAC1 oligomerization. Instead, it appears that VBIT-4 functions as a membrane-active compound.

Strengths:

This is a carefully conducted and well-written study that highlights potential side effects of VBIT-4, a compound that has been used to study the role of VDAC1 in a range of physiological and pathological conditions. The work is of interest to a broad readership by showcasing the importance of a systematic assessment of drug-membrane interactions to identify potential off-target membrane-driven effects of small molecules that may be mistakenly attributed to the inhibition of specific proteins. Its strength lies in the variety of complementary approaches the authors used to rigorously challenge the effect of VBIT-4 on VDAC1 organization and function. Overall, the experimental data are compelling and of high quality.

Weaknesses:

The authors used high-speed atomic force microscopy (HS-AFM) to study the impact of VBIT-4 on VDAC1 oligomerization in real time at nanoscale resolution. Toward this end, they adsorbed POPC:POPE:cholesterol membranes reconstituted with or without VDAC1 on mica. This revealed that addition of VBIT-4 produced small perforations in the bilayer that were independent of VDAC1. In the absence of VBIT-4, VDAC1 showed the characteristic honeycomb topography that the authors described in a previous study (Ref. 17). To quantitatively assess whether VBIT-4 affects VDAC1 organization, they analyzed protein compaction within clusters using inter-protein distance measurements. This analysis revealed no significant difference in VDAC1 organization between control conditions, 1 uM and 10 uM VBIT-4, supporting a model in which VBIT-4 primarily perturbs the lipid matrix rather than VDAC1 assemblies. This conclusion is based on the assumption that VDAC channels retain some lateral mobility in bilayers adsorbed onto mica, for which the manuscript does not provide direct evidence. In their rebuttal, the authors cite previous studies indicating that VDAC channels and beta-barrel proteins with larger extracellular domains exhibit measurable lateral diffusion in supported lipid bilayers formed on mica. Based on this, they conclude that their methodology does not constitute a limiting factor for lateral diffusion. It would be appropriate to cover this point and cite the corresponding references in the manuscript.

# Referee report: Reviewer #2 (Public review)

**DOI:** 10.7554/eLife.111980.3.sa2

Summary

This manuscript re-evaluates the mechanism of action of VBIT-4, a compound widely used as a putative inhibitor of VDAC1 oligomerization. The authors test whether VBIT-4 acts directly on VDAC1 assemblies or instead perturbs lipid membranes more generally. Using high-speed atomic force microscopy, electrophysiology, liposome leakage assays, Laurdan fluorescence, microscale thermophoresis, coarse-grained molecular dynamics simulations, and cell-based assays in wild-type and VDAC1-knockout HeLa cells, they show that VBIT-4 partitions into lipid bilayers, induces membrane defects and leakage, and causes VDAC1-independent cytotoxicity at concentrations commonly used in the literature to infer VDAC1-specific effects.

Strengths

The main strength of the study is the convergence of multiple independent approaches on the same central conclusion. Atomic force microscopy directly visualizes VBIT-4-induced defects in lipid regions while VDAC1 assemblies remain apparently intact. Electrophysiology separates VDAC1 channel behavior from background membrane conductance and shows that VBIT-4 does not measurably alter VDAC1 conductance or voltage gating, while increasing nonspecific membrane permeability. Lipid-only membranes, lipid nanodiscs lacking VDAC1, and VDAC1-knockout cells provide important controls supporting a VDAC1-independent mechanism.

The wild-type versus VDAC1-knockout cytotoxicity comparison is a particularly strong test of VDAC1 independence at concentrations above 10 µM. The manuscript also usefully emphasizes that VBIT-4 is poorly soluble, aggregation-prone, pH-dependent, membrane-partitioning, and storage-sensitive. These properties are important for interpreting variability across previous studies using this compound.

The manuscript is careful in defining the scope of its conclusions. It distinguishes AFM- and simulation-based measurements of VDAC1 cluster organization from cross-linking-defined proximity, which is important because these are related but non-equivalent readouts of VDAC1 organization. It also explicitly discusses how VBIT-4 solubility, aggregation, protonation, membrane partitioning, and storage sensitivity complicate comparisons based on nominal compound concentration. These points help readers interpret both the current data and the broader literature using VBIT-4.

Limitations

The cellular data strongly support VDAC1-independent cytotoxicity above 10 µM, but the lower-dose mitochondrial functional phenotypes, including effects on respiration, mitochondrial calcium, and mitochondrial membrane potential, were not directly compared between wild-type and VDAC1-knockout backgrounds. The manuscript appropriately avoids overinterpreting these mitochondrial effects as directly VDAC1-independent, but readers should note that VDAC1 independence is more firmly established for cytotoxicity than for the lower-dose mitochondrial phenotypes.

The coarse-grained simulations provide useful mechanistic support for membrane partitioning, aggregation, and defect formation. However, the partitioning validation relies on the neutral VBIT-4 species and comparison with empirical partition-coefficient predictors rather than a matched all-atom octanol-water transfer calculation using the same atomistic model. This is a reasonable modeling choice, but it does not eliminate the likely importance of atomistic-level details for accurately describing pore formation. This is especially relevant for a compound with pH-dependent protonation, aggregation, and interfacial membrane localization. The simulation-derived partitioning and pore-formation results should therefore be interpreted as strong qualitative and mechanistic support rather than as a definitive quantitative description of VBIT-4 behavior across all protonation states, concentrations, and membrane environments.

Overall assessment

Overall, this is an important and timely study that provides a strong reassessment of VBIT-4 as a tool compound. The evidence that VBIT-4 perturbs lipid membranes independently of VDAC1 is compelling and should be useful for researchers interpreting past and future studies that use VBIT-4 as a probe of VDAC1 function.

# Author comment: Author response

**DOI:** 10.7554/eLife.111980.3.sa3

**Authors:** Varun Ravishankar, Luís Borges-Araújo, Megha Rajendran, Elodie Lafargue, Deborah Byrne, Nicolas Buzhinsky, Mya S Wolfe, Wendy Fitzgerald, Nina A Bautista, Bethel G Beyene, Motahareh G Larimi, Jean-Pierre Duneau, James Sturgis, Sergey Bezrukov, Ignacio Casuso, Tatiana Rostovtseva, Lucie Bergdoll

**Affiliations:** Aix-Marseille Université Marseille France; Centre National de la Recherche Scientifique Lyon France; National Institute of Health Bethesda United States; Inserm MARSEILLE France; Institut de Microbiologie de la Méditerranée, CNRS Marseille France; Inserm MARSEILLE France; National Institute of Health Bethesda United States; National Institute of Health BEthesda United States; National Institute of Health Bethesda United States; National Institute of Health Bethesda United States; National Institute of Health Bethesda United States; Aix-Marseille Université Marseille France; Aix-Marseille Université Marseille France; National Institute of Health Bethesda United States; U1067 INSERM, Aix-Marseille Université Marseille France; National Institute of Health Bethesda United States; Centre National pour la Recherche Scientifique et Technique (CNRST) Marseille France

The following is the authors’ response to the original reviews.

**Public Reviews:**

**Reviewer #1 (Public review):**

The authors used high-speed atomic force microscopy (HS-AFM) to study the impact of VBIT-4 on VDAC1 oligomerization in real time at nanoscale resolution. Toward this end, they adsorbed POPC:POPE:cholesterol membranes reconstituted with or without VDAC1 on mica. This revealed that the addition of VBIT-4 produced small perforations in the bilayer that were independent of VDAC1. In the absence of VBIT-4, VDAC1 showed the characteristic honeycomb topography that the authors described in a previous study (Reference 17). To quantitatively assess whether VBIT-4 affects VDAC1 organization, they analyzed protein compaction within clusters using inter-protein distance measurements. This analysis revealed no significant difference in VDAC1 organization between control conditions, 1 uM and 10 uM VBIT-4, supporting a model in which VBIT-4 primarily perturbs the lipid matrix rather than VDAC1 assemblies. This conclusion is based on the assumption that VDAC channels retain some lateral mobility in bilayers adsorbed onto mica. Do the authors have evidence that this is indeed the case? Did they also perform HS-AFM on VDAC1-containing membranes treated with VBIT-4 prior to adsorption onto mica?

We thank the reviewer for this important question regarding the lateral mobility of VDAC1 in supported lipid bilayers (SLBs).

It is well established that membrane proteins can retain lateral mobility in SLBs formed on mica. This is enabled by the presence of a thin interstitial water layer (typically on the order of 10–20 Å) between the substrate and the bilayer, which reduces frictional coupling and preserves membrane fluidity. This property is a key advantage of SLB systems and has been extensively described [1].

Furthermore, HS-AFM studies provide experimental evidence supporting such mobility. For example, Casuso et al. [2] showed that OmpF trimers—whose extracellular domain is larger than that of VDAC1— exhibit measurable lateral diffusion in supported membranes. This indicates that even relatively bulky membrane proteins are not immobilized by the mica support.

In the specific case of VDAC1, our data provide direct evidence of mobility. As shown in Figure 4J of Ref. 17, individual VDAC1 pores display lateral displacements within the membrane, despite the presence of strong protein–protein interactions. This observation indicates that VDAC1 is not rigidly immobilized upon adsorption.

Regarding the reviewer’s question about VBIT-4 treatment prior to membrane adsorption onto mica, we also performed experiments in which VDAC1-containing proteoliposomes were pre-incubated with VBIT4 before deposition onto mica and formation of supported lipid bilayers. These samples were not imaged at sufficiently high resolution to allow the same quantitative analysis of VDAC1 cluster organization as performed for the experiments shown in the main text. However, at the resolution obtained, we did not observe obvious large-scale changes in membrane organization compared with samples in which VBIT-4 was added after supported bilayer formation.

Finally, in light of VDAC1 mobility observed under our experimental conditions and the well-established properties of SLBs, the mica support does not constitute a major limiting factor for lateral diffusion.

**Reviewer #2 (Public review):**

(1) The main limitation is that the conclusion that VBIT-4 does not affect VDAC1 oligomerization is strongest for the specific readouts used here: atomic force microscopy measurements of cluster compaction, VDAC1 channel properties, and simulated assembly behavior. These are direct and informative measurements, but they are not identical to the chemical cross-linking readouts used in much of the prior VBIT-4 literature. Readers should therefore distinguish between VDAC1 cluster organization in membranes, as measured here, and cross-linking-defined VDAC1 proximity.

We agree with the reviewer that AFM-defined VDAC1 cluster organization and cross-linking-defined VDAC1 proximity are related but non-equivalent readouts, and we have revised the manuscript to make this distinction explicit. However, this distinction also highlights an important limitation in interpreting changes in cross-linking efficiency as direct evidence of altered VDAC1 oligomerization. Chemical cross-linking primarily reports the proximity and accessibility of reactive residues and does not directly provide information on the number, size, stability, or supramolecular organization of VDAC1 assemblies. Moreover, VDAC1 organization is strongly influenced by the lipid environment, as shown in giant proteoliposomes with reconstituted VDAC1 by fluorescence correlation spectroscopy [3] and AFM [4], and changes in protein spacing or orientation within dynamic lipid–protein clusters could alter cross-linking efficiency without disrupting the assemblies themselves.

Cross-linking can provide valuable information when interpreted in the context of independently defined oligomeric structures or interfaces, as recently illustrated by Takeda et al. for yeast Por1 [5]. However, in most studies reporting inhibition of VDAC1 oligomerization by VBIT-4, the evidence relies primarily on SDSPAGE analysis of chemically cross-linked species, frequently quantified as changes in VDAC1 dimers. In contrast, our HS-AFM measurements directly assess the spatial organization and compaction of VDAC1 assemblies in lipid membranes, while our simulations independently assess assembly behavior. Although these approaches do not measure cross-linking efficiency, neither reveals measurable disruption of VDAC1 assemblies by VBIT-4 under the conditions tested. Our observations are therefore difficult to reconcile with the interpretation that VBIT-4 inhibits VDAC1 assembly formation. Instead, we propose that previously reported changes in cross-linking efficiency could reflect changes in protein proximity, orientation, or residue accessibility within dynamic lipid–protein clusters, particularly given the membrane-perturbing properties of VBIT-4 demonstrated here, rather than disruption of VDAC1 assemblies themselves.

We have therefore revised the Discussion to acknowledge that these approaches probe distinct aspects of VDAC1 organization, while also clarifying that changes in cross-linking efficiency alone cannot be interpreted as direct evidence that VBIT-4 inhibits VDAC1 assembly formation.

“Cross-linking—the assay most commonly used to monitor VDAC1 “oligomerization”—does not report on oligomer number or stability but rather on the proximity of proteins within these adaptable clusters in MOM. In contrast, HS-AFM directly measures the spatial organization of VDAC1 assemblies in lipid membranes, while molecular dynamics simulations provide an independent description of assembly behavior. These approaches therefore probe related, but non-equivalent, aspects of VDAC1 organization. The organization of VDAC1 in the MOM is extremely sensitive to lipid composition [17]; any hydrophobic compound that perturbs membrane properties may therefore influence cross-linking efficiency through changes in protein spacing, orientation, or residue accessibility, without necessarily altering the overall organization of VDAC1 assemblies. Accordingly, although our results do not directly address cross-linking efficiency, AFM quantification (Supplementary Figure 2) and molecular dynamics simulations (Supplementary Figure 8) consistently show that VBIT-4 does not measurably alter VDAC1 cluster compaction or prevent assembly formation under the conditions examined.”

(2) A second limitation is the uncertainty around effective VBIT-4 concentration. Because VBIT-4 is poorly soluble, aggregation-prone, pH-dependent, membrane-partitioning, and storage-sensitive, nominal added concentration may differ substantially from the concentration of active compound available in each assay. This complicates comparisons across the different in vitro, simulation, cellular, and previously published assays.

We thank the reviewer for this important comment. We agree that the nominal concentration of VBIT-4 does not necessarily reflect the effective concentration of active compound available in solution or within lipid membranes. We have therefore expanded the Discussion to explicitly distinguish nominal from effective concentration and to emphasize that, because of VBIT-4's poor solubility, aggregation, membrane partitioning, pH-dependent protonation, and limited stability during storage, the effective concentration cannot be readily determined or compared across experimental systems. “As a consequence, the nominal concentration of VBIT-4 added to an experiment is unlikely to correspond to the effective concentration of active compound available in solution or within lipid membranes. The effective concentration is expected to vary substantially with pH, storage conditions, formulation, and membrane composition, complicating direct comparisons between different assays and across studies. “

Importantly, to facilitate comparison with the existing literature, we deliberately used the same nominal concentration range as previous studies investigating VBIT-4. Thus, although the effective membrane concentration is inherently uncertain, this limitation applies equally to previous studies using VBIT-4 and represents an intrinsic limitation of the compound rather than of our experimental approach. Measuring the effective membrane concentration is currently not feasible and is beyond the scope of the present study. We further note that this intrinsic uncertainty likely contributes to the variability observed between published studies.

(3) The coarse-grained simulations provide a coherent mechanistic framework for membrane partitioning, aggregation, and defect formation. However, the VBIT-4 coarse-grained model is newly parameterized and is used to support a quantitative partitioning argument. The manuscript would be easier to interpret if the coarse-grained-derived partition coefficient were reported with uncertainty, convergence information, and protonation state, and compared with a matched all-atom octanol-water partition estimate from the same atomistic model used to build the coarse-grained mapping. This matters because the partitioning argument is used quantitatively to relate micromolar aqueous VBIT-4 to millimolar concentrations in the bilayer.

Following the Reviewer’s comments, we have expanded the Methods section to add further detail and references on the transfer free-energy calculations and include the requested details. The protonation state used throughout is neutral VBIT, as alchemical free energy calculations of charged solutes require additional corrections, and reliable reference logP values for charged species are scarce given that most empirical predictors are parameterised for neutral molecules; this is the standard Martini pathway for nonbonded term validation.

The calculated octanol/water logP for neutral VBIT, averaged over three independent replicates, is 3.52 ± 0.01 (replicate values: 3.54, 3.53, 3.51). Convergence and overlap diagnostics confirmed well-sampled simulations across all lambda windows; the corresponding forward/backward convergence plots and MBAR overlap matrices are provided in the Supporting Information.

Regarding the suggestion to compare against a matched atomistic free-energy calculation: we chose not to pursue this route, as atomistic MD-based logP estimates are not a more reliable reference than empirical predictors for this purpose. Benchmark studies have shown that empirical consensus methods generally outperform atomistic free-energy calculations when compared against experiment, while being substantially less computationally demanding (See [6,7]). We therefore benchmark against a consensus of five established empirical logP predictors (iLOGP, XLOGP3, WLOGP, MLOGP, SILICOS-IT via SwissADME), yielding a consensus logP of 3.42 ± 0.63 for neutral VBIT, in good agreement with our CG estimate of 3.52 ± 0.01.

We replaced the following manuscript text in the Methods section:

“These choices were validated by estimating CG octanol/water partitioning free energies, which were compared to predictors obtained via SwissADME[80] (iLOGP[81], XLOGP3[82], WLOGP[83], MLOGP[84], SILICOS-IT). The calculated partitioning free energies were obtained by thermodynamic integration as described elsewhere[77].”

by the following:

“These choices were validated by calculating CG octanol/water partitioning free energies for neutral VBIT and comparing them against a consensus of reference values obtained by theoretical predictors. Rather than comparing our Martini logP measurements against atomistic molecular dynamics calculations, we benchmark against established logP prediction methods. For equilibrium octanol/water partitioning, empirical predictors have generally demonstrated accuracy comparable to, or better than, atomistic free energy calculations when evaluated against experiment [6]. We further use a consensus of five empirical models, as consensus predictions have been shown to outperform individual predictors [7]. Reference logP values for neutral VBIT were obtained using the prediction methods available through SwissADME [8] (iLOGP [9], XLOGP3 [10], WLOGP [11], MLOGP [12], SILICOS-IT), yielding predicted logP values of 3.66, 3.85, 4.06, 2.25, and 3.28, respectively. The average of these predictions was used as a consensus estimate, giving a logP value of 3.42 ± 0.63.”

“The calculated CG partitioning free energies were obtained for neutral VBIT by thermodynamic integration as described elsewhere [13]. In short, the solute is alchemically decoupled from each solvent environment independently across 12 lambda windows, gradually turning off all non-bonded interactions between the solute and its surroundings. The free energy change along this path corresponds to the solvation free energy in that solvent, and taking the difference between octanol and water (ΔG_octanol − ΔG_water) yields the transfer free energy, which is converted to a partition coefficient. Three independent replicates were run, yielding octanol-water logP values of 3.54, 3.53, and 3.51, with an average of 3.52 ± 0.01. Convergence and overlap analysis confirmed that all lambda windows were well-sampled and that the free energy estimates were statistically reliable as required per the guidelines for the analysis of free energy calculations [14]; the corresponding forward/backward convergence plots and MBAR overlap matrices are provided in the Supplementary Figures 11 and 12.”

(4) Finally, the cellular data strongly support VDAC1-independent cytotoxicity, but the lower-dose mitochondrial functional phenotypes were not directly compared between wild-type and VDAC1-knockout backgrounds. VDAC1 independence is therefore more directly established for cytotoxicity than for the lower-dose mitochondrial phenotypes.

We agree that our cellular data cannot confirm that the mitochondrial effect of VBIT-4 is independent of VDAC1. However, a previous study by Belosludtsev et al. shows a similar decrease in membrane potential upon VBIT-4 treatment due to inhibition of electron transport chain complexes [15]. Following the Reviewer’s advice, we narrowed the wording accordingly in the Results and Discussion sections.

Overall, this work provides a valuable and timely reassessment of VBIT-4, and its central conclusion will be useful for researchers interpreting studies that use this compound as a probe of VDAC1 function.

Suggestions for authors:

(1) Soften categorical statements such as "VBIT-4 does not alter VDAC1 oligomerization" by specifying the tested readouts: VDAC1 cluster compaction, channel properties, and simulated assembly behavior under the conditions used here. A matched cross-linking experiment under the authors' own VBIT-4 handling and concentration conditions could be useful, but is not essential; the essential point is to make clear that cross-linking-defined VDAC1 proximity and AFM/simulation-defined membrane cluster organization are related but non-equivalent readouts.

We modified the manuscript to soften the tone. In the discussion, we now emphasize that cross-linking and HS-AFM/simulation probe related but non-equivalent aspects of VDAC1 organization, and that differences in cross-linking efficiency previously reported could be due to alteration of protein spacing or orientation.

These findings demonstrate that VBIT-4 acts by perturbing lipid bilayers rather than through detectable direct modulation of VDAC1,

Change title: VBIT-4 Does Not Alter VDAC1 Oligomerization

To “VBIT-4 Does Not Measurably Alter VDAC1 Cluster Organization or Assembly Behaviour”

This indicates that VBIT-4 neither prevents nor disrupts VDAC oligomerization.

To “This indicates that VBIT-4 does not measurably prevent or disrupt VDAC1 assembly under the simulated conditions.”

“Cross-linking—the assay most commonly used to monitor VDAC1 “oligomerization”—does not report on oligomer number or stability but rather on the proximity of proteins within these adaptable clusters in MOM. In contrast, HS-AFM directly measures the spatial organization of VDAC1 assemblies in lipid membranes, while molecular dynamics simulations provide an independent description of assembly behavior. These approaches therefore probe related, but non-equivalent, aspects of VDAC1 organization. The organization of VDAC1 in the MOM is extremely sensitive to lipid composition [17]; any hydrophobic compound that perturbs membrane properties may therefore influence cross-linking efficiency through changes in protein spacing, orientation, or residue accessibility, without necessarily altering the overall organization of VDAC1 assemblies. Accordingly, although our results do not directly address cross-linking efficiency, AFM quantification (Supplementary Figure 2) and molecular dynamics simulations (Supplementary Figure 8) consistently show that VBIT-4 does not measurably alter VDAC1 cluster compaction or prevent assembly formation under the conditions examined.”

(2) More explicitly distinguish nominal added VBIT-4 concentration from effective available concentration, given the solubility, aggregation, pH-dependence, membrane partitioning, and storagesensitivity observations.

We modified the Discussion (see public review)

(3) Report the coarse-grained-derived octanol/water partition coefficient or transfer free energy numerically, with uncertainty, convergence information, and protonation state. Consider providing the corresponding all-atom octanol/water transfer free energy or partition coefficient for the same protonation state(s).

We answered this comment and added two Supplemental figures 11 and 12. (see public review)

(4) Either repeat the oxygen consumption rate, TMRM, and Rhod-2 assays in VDAC1-knockout cells, or narrow the wording so that only cytotoxicity is described as directly shown to be VDAC1-independent.

Thank you for pointing this out. Following the Reviewer’s advice, we narrowed the wording accordingly in the Results (suppression of “This demonstrates that the cytotoxicity is due to a loss of membrane integrity.”) and Discussion sections.

We removed the direct link to VDAC1: At concentrations below 10 μM, VBIT-4 decreased mitochondrial calcium, respiration, and membrane potential in HeLa cells without affecting mitochondrial mass. They align with reports that VBIT-4 also accumulates in the mitochondrial inner membrane, where it inhibits respiratory complexes I, III, and IV and decreases mitochondrial membrane potential [15,16].

(5) Consider moving the storage-stability observation into the main text, given its likely importance for interpreting variability in the broader VBIT-4 literature. It would also be useful to include clearer information on stock age, storage temperature, freeze-thaw history, solvent conditions, and whether precipitation or turbidity was observed.

The Supplemental Figure 9C was moved the main text as new Figure 6, and additional information about storage conditions is added to the Methods section.

(6) Minor correction: the parenthetical "10^3.5 = 3.2" should be corrected. Since 10^3.5 is approximately 3,162, the intended statement appears to be that a 1 µM aqueous concentration corresponds to approximately 3.2 mM in the bilayer.

Thank you for pointing it out, it is corrected.

References:

(1) Castellana, E. T. & Cremer, P. S. Solid supported lipid bilayers: From biophysical studies to sensor design. Surf. Sci. Rep. 61, 429–444 (2006).

(2) Casuso, I. et al. Characterization of the motion of membrane proteins using high-speed atomic force microscopy. Nat. Nanotechnol. 7, 525–529 (2012).

(3) Betaneli, V., Petrov, E. P. & Schwille, P. The role of lipids in VDAC oligomerization. Biophys. J. 102, 523–531 (2012).

(4) Lafargue, E. et al. Lipid composition of the membrane governs the oligomeric organization of VDAC1. 2024.06.26.597124 Preprint at https://doi.org/10.1101/2024.06.26.597124 (2024).

(5) Takeda, H. et al. Oligomer-based functions of mitochondrial porin. Nat. Commun. 16, (2025).

(6) Işık, M. et al. Assessing the accuracy of octanol–water partition coefficient predictions in the SAMPL6 Part II log P Challenge. J. Comput. Aided Mol. Des. 34, 335–370 (2020).

(7) Calculation of molecular lipophilicity: State‐of‐the‐art and comparison of log P methods on more than 96,000 compounds - Mannhold - 2009 - Journal of Pharmaceutical Sciences - Wiley Online Library. https://onlinelibrary.wiley.com/doi/10.1002/jps.21494.

(8) Daina, A., Michielin, O. & Zoete, V. SwissADME: a free web tool to evaluate pharmacokinetics, druglikeness and medicinal chemistry friendliness of small molecules. Sci. Rep. 7, 42717 (2017).

(9) Daina, A., Michielin, O. & Zoete, V. iLOGP: a simple, robust, and efficient description of noctanol/water partition coefficient for drug design using the GB/SA approach. J. Chem. Inf. Model. 54, 3284–3301 (2014).

(10) Cheng, T. et al. Computation of octanol-water partition coefficients by guiding an additive model with knowledge. J. Chem. Inf. Model. 47, 2140–2148 (2007).

(11) Wildman, S. A. & Crippen, G. M. Prediction of Physicochemical Parameters by Atomic Contributions. J. Chem. Inf. Comput. Sci. 39, 868–873 (1999).

(12) Lipinski, C. A., Lombardo, F., Dominy, B. W. & Feeney, P. J. Experimental and computational approaches to estimate solubility and permeability in drug discovery and development settings. Adv. Drug Deliv. Rev. 46, 3–26 (2001).

(13) Souza, P. C. T. et al. Protein-ligand binding with the coarse-grained Martini model. Nat. Commun. 11, 3714 (2020).

(14) Klimovich, P. V., Shirts, M. R. & Mobley, D. L. Guidelines for the analysis of free energy calculations. J. Comput. Aided Mol. Des. 29, 397–411 (2015).

(15) Belosludtsev, K. N. et al. Effect of VBIT-4 on the functional activity of isolated mitochondria and cell viability. Biochim. Biophys. Acta Biomembr. 1866, 184329 (2024).

(16) Belosludtsev, K. N. et al. Pharmacological and Genetic Suppression of VDAC1 Alleviates the Development of Mitochondrial Dysfunction in Endothelial and Fibroblast Cell Cultures upon Hyperglycemic Conditions. Antioxid. Basel Switz. 12, 1459 (2023).
